# A Deep-Learning Empowered, Real-Time Processing Platform of fNIRS/DOT for Brain Computer Interfaces and Neurofeedback

**DOI:** 10.1109/TNSRE.2025.3553794

**Published:** 2025-03-21

**Authors:** Yunjia Xia, Jianan Chen, Jinchen Li, Tingchen Gong, Ernesto E. Vidal-Rosas, Rui Loureiro, Robert J. Cooper, Hubin Zhao

**Affiliations:** HUB of Intelligent Neuro-engineering (HUBIN), CREATe, Division of Surgery and Interventional ScienceUniversity College London (UCL) London WC1E 6BT U.K.; School of Electronics and Computer ScienceUniversity of Southampton 7423 Southampton SO17 1BJ U.K.; DOT-HUB, Department of Medical Physics and Biomedical EngineeringUCL London WC1E 6BT U.K.

**Keywords:** Functional near-infrared spectroscopy (fNIRS), diffuse optical tomography (DOT), brain-computer interface (BCI), neurofeedback (NFB), motion artifacts, deep learning, real-time processing

## Abstract

Brain-Computer Interfaces (BCI) and Neurofeedback (NFB) approaches, which both rely on real-time monitoring of brain activity, are increasingly being applied in rehabilitation, assistive technology, neurological diseases and behavioral disorders. Functional near-infrared spectroscopy (fNIRS) and diffuse optical tomography (DOT) are promising techniques for these applications due to their non-invasiveness, portability, low cost, and relatively high spatial resolution. However, real-time processing of fNIRS/DOT data remains a significant challenge as it requires establishing a baseline of the measurement, simultaneously performing real-time motion artifact (MA) correction across all channels, and (in the case of DOT) addressing the time-consuming process of image reconstruction. This study proposes a real-time processing system for fNIRS/DOT that integrates baseline calibration, denoising autoencoder (DAE) based MA correction model with a sliding window strategy, and a pre-calculated inverse Jacobian matrix to streamline the reconstructed 3D brain hemodynamics. The DAE model was trained on an extensive whole-head high-density DOT (HD-DOT) dataset and tested on separate motor imagery dataset augmented with artificial MA. The system demonstrated the capability to simultaneously process approximately 750 channels in real-time. Our results show that the DAE-based MA correction method outperformed traditional MA correction in terms of mean squared error and correlation to the known MA-free data while maintaining low latency, which is critical for effective BCI and NFB applications. The system’s high-channel, real-time processing capability provides channel-wise oxygenation information and functional 3D imaging, making it well-suited for fNIRS/DOT applications in BCI and NFB, particularly in movement-intensive scenarios such as motor rehabilitation and assistive technology for mobility support.

## I. Introduction

Brain-computer interfaces (BCIs) and neurofeedback (NFB), which leverage real-time feedback on brain activity, have been widely utilized in rehabilitation, assistive technology, neurological diseases and behavioral disorders [1], [2], [3], [4], [5]. BCI is a direct communication pathway that measures, decodes, and translates electrical, magnetic, or metabolic brain activity into commands for controlling external devices [2]. NFB is a form of biofeedback that trains individuals to self-regulate brain functions by measuring their brain activities and providing feedback signals [6]. Real-time processing is critical for effective BCIs and NFB applications. In BCIs, real-time processing supports user interaction with external devices by interpreting neural signals to execute specific commands, providing feedback to refine control and improve system performance. In NFB, it provides users with timely feedback on their brain activity, enabling self-regulation and behavioral adjustments. The real-time capability not only facilitates seamless interaction but also promotes adaptive learning by enabling task-specific neural activation in motor rehabilitation and reinforcing targeted cognitive processes in cognitive trainings [7].

Techniques such as electroencephalography (EEG), functional magnetic resonance imaging (fMRI), and functional near-infrared spectroscopy (fNIRS) are commonly used to monitor brain activity in BCI/NFB [8]. EEG is a widely used modality in real-time BCI/NFB mainly due to its high temporal resolution [2]. By recording the electrical signals generated by en-masse neuronal activity with millisecond temporal precision, EEG is ideal for the rapid detection of neural signals. On the other hand, EEG has relatively low spatial resolution and is highly susceptible to motion and electrophysiological artifacts.

To remedy these situations and potentially complement with EEG, fNIRS has been utilized in BCI and NFB applications due to its higher spatial resolution and potential higher tolerance to motion artifacts (MAs). fNIRS works by emitting near-infrared light that propagates through the skin and skull to the brain. A proportion of this light is scattered back to the surface, carrying with it information about the concentration changes of oxygenated (HbO) and deoxygenated hemoglobin (HbR) in the cortical regions, which are closely linked to local neuronal activity. Diffuse Optical Tomography (DOT) extends the principles of fNIRS by employing an array of multiple near-infrared light sources and detectors at different source-detector separations, enabling overlapping spatial sampling to reconstruct 3D images of cortical hemodynamic activity. It offers enhanced spatial resolution and depth sensitivity compared to conventional fNIRS [9]. High-density DOT (HD-DOT) refines DOT by utilizing a denser source-detector array, spanning short distances (<15 mm) to long distances ( $\ge 30$ mm), and an optode density of ~0.5 to 2 cm^−2^ or more [10]. This configuration significantly increases spatial sampling, enhancing resolution and depth specificity while minimizing extracerebral contamination [11]. The low cost, fine wearability, and relatively high spatial resolution of these optical methods, make them highly effective for functional neuroimaging. These qualities also make fNIRS, DOT and HD-DOT ideal complements to EEG-based BCI/NFB by providing the spatial resolution that EEG lacks [12]. The enhanced spatial resolution can be used to better target brain regions and networks with improved anatomical precision beyond EEG [7].

However, real-time processing of fNIRS/DOT signals faces several challenges. The commonly used continuous-wave fNIRS/DOT measures only the intensity changes of near-infrared light after it travels through tissue, allowing it to capture relative changes in hemoglobin concentrations [13]. As a result, each experiment requires a baseline, which can be difficult to establish in real-time measurements.

Additionally, raw fNIRS/DOT signals are often contaminated by various noise sources, including MAs, systemic physiological fluctuations, and low-frequency oscillations [14]. Extracting accurate cortical hemodynamic information requires some complex and time-consuming preprocessing steps. While offline analysis can address these issues by leveraging the complete experimental dataset, real-time processing is hindered by the absence of prior information and potential delays. Recent advances in deep learning have revolutionized noise suppression for neuroimaging modalities like EEG, fMRI, and fNIRS/DOT [15], [16], [17], [18], [19]. Kim et al. employed a U-Net architecture to reconstruct the hemodynamic response linked to neuronal activity while reducing MA [18]. Gao et al. utilized a denoising autoencoder (DAE) to refine preprocessed hemodynamic response signals by suppressing residual MAs [19]. These deep learning approaches offer distinct advantages over traditional methods, such as automated feature extraction (eliminating the manual parameter tuning), robustness to non-stationary noise, and end-to-end preservation of functional signals while suppressing artifacts. However, in fNIRS/DOT, the real-time performance of deep-learning-based denoising for multi-channel systems remains underexplored. Existing studies often focus on offline processing or single-channel signals, neglecting the computational latency and synchronization challenges inherent to real-time, DOT and HD-DOT applications.

For DOT and HD-DOT, generating 3D images requires image reconstruction [10]. This process involves computing and inverting a Jacobian matrix that describes the correlation between changes in tissue absorption coefficients and the changes in detected optical intensity at each channel. This process is computationally intensive. These challenges impede the ability of fNIRS, particularly DOT and HD-DOT, to monitor neural activity in real-time, this can compromise the interpretation of neural signals using fNIRS/DOT in BCI and hinder the reliability of the feedback in NFB.

**Fig. 1.**
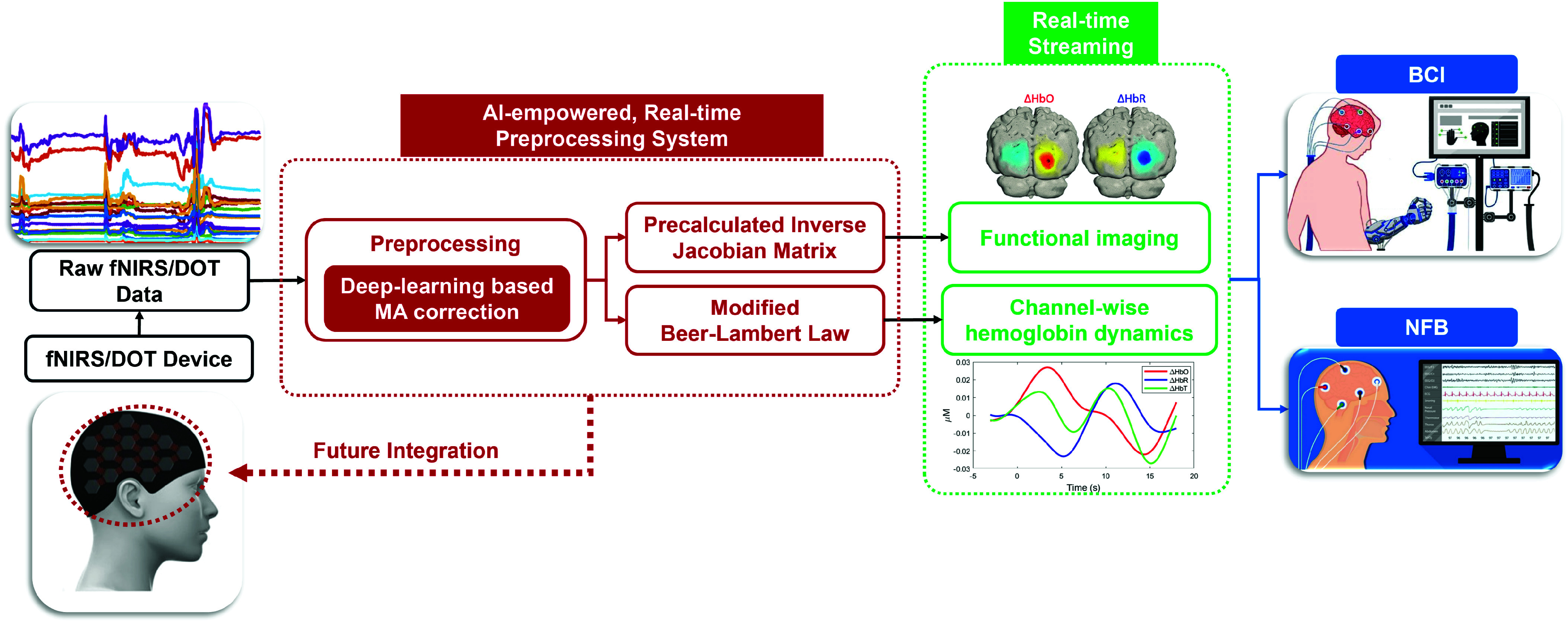
Overview of the proposed AI-empowered real-time, processing platform of fNIRS/DOT.

To address these challenges, we propose a real-time fNIRS/DOT processing system incorporating a deep-learning-based MA correction module, as shown in Fig. 1. The system includes a calibration process to establish a baseline for real-time measurements, employs a DAE model to correct MA, and applies a sliding window strategy for real-time MA correction. Additionally, a pre-calculated inverse Jacobian matrix is utilized to enable efficient real-time image reconstruction for DOT and HD-DOT systems. The main contributions of this paper include:

• Investigating a real-time processing framework for fNIRS/DOT that incorporates advanced deep-learning techniques for MA correction.
• Proposing a sliding window strategy to enable real-time application of the deep-learning model for MA correction, ensuring accurate and dynamic data acquisition of hemodynamic responses.
• Introducing a pre-calculated inverse Jacobian matrix to streamline and accelerate 3D image reconstruction for DOT and HD-DOT systems.
• Validating the effectiveness of the system in providing real-time channel-based hemodynamic data and high-resolution 3D hemodynamic imaging, addressing key limitations in real-time fNIRS/DOT processing, particularly for applications in BCI and NFB.

## II. Background

**Fig. 2.**
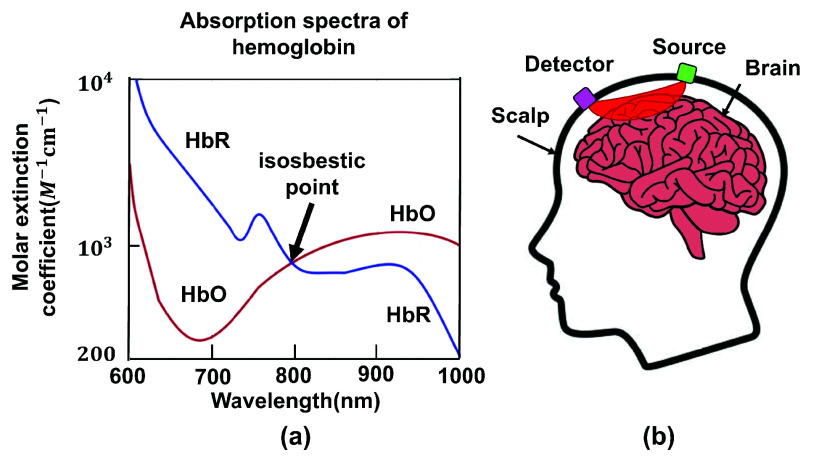
a) The absorption spectra of Oxyhaemoglobin (HbO) and Deoxyhaemoglobin (HbR) in the Near-Infrared Wavelength Range (650 to ~1000 nm). fNIRS systems typically operate at two wavelengths, usually with one above and one below the isosbestic point (808nm), at which HbO and HbR demonstrate the same absorption coefficient; b) Illustration of the source-detector pair in an fNIRS system, depicting the light path.

fNIRS and DOT utilize the distinct absorption spectra of HbO and HbR, the key molecules responsible for transporting oxygen in the bloodstream, within the near-infrared wavelength range (Fig. 2a). As illustrated in Fig. 2b, a near-infrared light source and a detector are placed on the scalp, forming an optical channel. Changes in light intensity through the scalp and brain tissue are used to infer HbO and HbR concentration variations, enabling precise tracking of cerebral hemodynamics and brain activity via the hemodynamic response function (HRF) during various tasks [13].

The typical fNIRS/DOT imaging pipeline includes preprocessing, forward modeling, and inverse problem solving. The raw data obtained from the fNIRS/DOT device represents an arbitrarily scaled light intensity signal for each channel. This includes channel pruning, where channels with low signal-to-noise ratios (SNR) or exhibiting saturation issues are removed. The SNR is calculated as the ratio of the standard deviation ( $std(I_{dB}))$ to the mean value of the light intensity $mean(I_{dB})$, expressed in decibels (dB) (shown in (1), (2)). The remaining channels are marked as active channels and are retained for further processing.

\begin{align*} I_{dB}& =10\times log10(I) \tag {1}\\ SNR& =\frac {std(I_{dB})}{mean(I_{dB})} \tag {2}\end{align*}

Subsequently, the detected light intensity is converted to optical absorbance (in units of optical density (OD)) using (3), where $I(t)$ represents the raw light intensity at time *t*, and $I_{baseline}$ denotes the baseline light intensity of the channel, typically calculated as the average intensity across the entire duration of the experiment.

\begin{equation*} OD= -\ln {\left ({{\frac {I(t)}{I_{baseline}}}}\right )} \tag {3}\end{equation*}

After OD conversion, MAs in each channel are corrected using various motion correction techniques. MAs typically result from subject movements during data collection – such as head movements that decouple the source/detector optodes from the scalp, leading to high-frequency spikes and baseline intensity shifts in the measured signals [14]. Accurate estimation of the hemodynamic response function in the channel requires the detection and removal of these MAs. Commonly used methods for MA correction include movement artifact reduction algorithm (MARA), wavelet-based filtering, temporal principal component analysis (tPCA), and temporal derivative distribution repair (TDDR) [20], [21], [22], [23], [24], [25].

Following MA correction, bandpass filtering is applied to eliminate physiological noise sources, such as cardiac pulse, respiration, and Mayer waves [26]. The filtered channel data is then used either for calculating HbO and HbR concentration changes ( $\Delta $HbO and $\Delta $HbR) at the channel level through the Modified Beer-Lambert Law, or for 3D image reconstruction of brain hemodynamics. For the channel-level hemodynamics calculation, the Modified Beer-Lambert Law is applied as shown in (4). In this equation, $\mathrm {\Delta }OD_{\lambda }$ represents the change of OD of the channel at different wavelengths $\lambda $, $a_{HbO}(\lambda)$and $a_{HbR}(\lambda)$ denote the specific absorption coefficient of oxyhemoglobin and deoxyhemoglobin at wavelength $\lambda $. The variable *x* is the source-detector separations and $D_{\lambda }$ is the estimated differential path-length factor at wavelength $\lambda $.

\begin{align*} \left [{{\begin{array}{cccccccccccccccccccc} \Delta HbO \\ \Delta HbR \\ \end{array}}}\right ]=\left [{{\begin{array}{cccccccccccccccccccc} a_{HbO}\left ({{ \lambda _{1} }}\right ) & a_{HbR}\left ({{ \lambda _{1} }}\right ) \\ a_{HbO}\left ({{ \lambda _{2} }}\right ) & a_{HbR}\left ({{ \lambda _{2} }}\right ) \\ \end{array}}}\right ]^{-1}\times \left [{{\begin{array}{cccccccccccccccccccc} \frac {\Delta {OD}_{\lambda _{1}}}{D_{\lambda _{1}}\cdot x} \\ \frac {\Delta {OD}_{\lambda _{2}}}{D_{\lambda _{2}}\cdot x} \\ \end{array}}}\right ] \tag {4}\end{align*}

For reconstructing 3D brain hemodynamics, forward modeling and inverse problem solving are two key steps. In forward modeling, the optode positions relative to the subject’s cranial landmarks are first collected. A Jacobian sensitivity matrix is then generated based on an appropriate model of photon transport, knowledge of the optode positions, and a 3D head model obtained from either the subject’s MRI scan or modified from a template MRI model if the subject’s MRI is unavailable. The Jacobian matrix describes the relationship between changes in optical absorption coefficient in the brain and the measured changes in OD.

To map the measured OD changes to brain hemodynamics, an inverse problem is solved using regularization techniques to acquire the inverse of the Jacobian matrix ( $\mathrm {J}^{-1}$). This inverse matrix is then multiplied by the change in OD to obtain the 3D optical changes, as expressed in (5):

\begin{equation*} \mathrm {J}^{-1}\cdot \Delta y=\Delta x \tag {5}\end{equation*}

where $\Delta y$ represents the change in measured optical parameters (in this case, the denoised OD), $\Delta x$ represents changes in the optical properties of tissue (in this case, the concentration changes of HbO and HbR) and $J^{-1}$ is the inverse Jacobian matrix. The result $\mathrm {\Delta }x$ can be used to create volume-wise images of 3D brain hemodynamics.

Achieving real-time fNIRS/DOT processing involves overcoming significant challenges at each stage of the processing. In the preprocessing stage, the absence of a channel baseline complicates the OD conversion, making it difficult to establish an accurate reference point for signal changes. Additionally, real-time correction of MAs is inherently complex due to the variability and unpredictability of motion.

Several studies have proposed solutions for baseline requirements and MA correction in fNIRS systems. In 2016, SyntBarker et al. introduced a modified linear Kalman filter for real-time MA correction [27]. In 2017, Lührs and Goebel developed a real-time fNIRS processing system that calculated channel baselines using the first 200 fNIRS values, implemented bandpass filtering, and employed pre-calculated Modified Beer-Lambert Law parameters [28]. This system also incorporated a MA correction method based on the negative correlation of HbO and HbR signals [29], alongside an incremental recursive least-squares procedure for real-time general linear model calculation. This system has been used for various NFB and BCI [30], [31], [32], [33], [34]. In 2022, Ortega-Martinez et al. proposed using a Kalman filter with time-embedded canonical correlations to distinguish brain signals from non-brain signals in real time [35]. In 2023, Anaya et al. presented an fNIRS system that achieved real-time preprocessing, including channel pruning, OD conversion, bandpass filtering, short-channel regression, and Modified Beer-Lambert Law [36].

Despite these advancements, real-time processing and imaging for DOT and especially HD-DOT systems have not been widely explored. The high channel count in these systems presents unique challenges. Real-time MA correction methods designed for fNIRS systems often face increased processing times and computational demands as the number of measurement channels grows, directly impacting the real-time processing and imaging of DOT and HD-DOT systems. This scaling issue hinders the effective application of these advanced imaging systems in real-time contexts.

Moreover, real-time processing for DOT and HD-DOT systems faces additional obstacles in the forward modeling and inverse problem-solving stages. The absence of optode positioning data introduces significant difficulties in achieving precise real-time 3D imaging of brain hemodynamics [8]. These combined challenges highlight the need for innovative approaches to enhance the real-time capabilities of DOT and HD-DOT systems in order to fully unblock their potential for applications in BCI and NFB.

## III. Methods

### A. Data Collection

Two separate datasets were employed: one to train the deep-learning-based MA correction model and the other to assess the performance of the proposed real-time system. All datasets were collected using the LUMO (Gowerlabs [37]), a wearable HD-DOT system with two different configurations tailored to their corresponding experiments and applications.

The first dataset was collected from 9 subjects wearing a 36-module wearable whole-head HD-DOT LUMO (Gowerlabs [37]). This device provides over 2500 effective channels with a signal-to-noise ratio (SNR) >17.8 dB across the whole head at two wavelengths (735 and 850 nm), with source-detector separations ranging from 10 mm to 60 mm. The participants underwent a range of tasks and stimuli, including resting, visual stimuli, somatosensory stimuli, auditory stimuli, finger tapping, and verbal fluency tasks, with an average experiment duration of 45 minutes per subject. MAs were manually generated and introduced into this dataset, creating paired noisy and clean data for training the DAE model. The high channel density covering the entire head and the diverse tasks included in this dataset enabled the model to capture the characteristics of fNIRS/DOT data across a wide range of conditions over the whole-head region. The data from 9 subjects was divided into training, validation, and test sets in a 6:1:2 ratio on a subject-wise basis to prevent self-learning. The test set was then processed using various MA correction techniques, including the proposed deep-learning method, to evaluate its effectiveness in correcting MAs.

The second dataset involved a motor imagery experiment, a commonly implemented task in BCI and NFB applications. Data was collected from a single subject wearing a 12-module wearable HD-DOT device (LUMO, Gowerlabs [37]), configured to cover the motor cortex region. This configuration provided 756 channels with an SNR >17.8 dB at two wavelengths, with source-detector separations ranging from 10 mm to 40 mm. The single-subject design was chosen to focus on demonstrating the core functionalities of the proposed processing system, including real-time MA correction across multiple channels and 3D hemodynamic image reconstruction. The subject performed motor imagery tasks, imagining squeezing a ball with either the left or right hand for 12 seconds per session, with 10 repetitions per hand and a 15-second rest between tasks. Synthetic MAs were manually generated and introduced into the dataset, which was subsequently processed using various MA correction techniques including MARA, tPCA, k-wavelet, splineSG, TDDR and the proposed real-time processing system [20], [21], [22], [23], [24], [25], to evaluate their effectiveness in mapping functional activities from MA-contaminated data.

### B. MA Simulation

To synthesize realistic MAs in fNIRS/DOT data, an experiment was conducted with a subject wearing a 12-module version of the wearable HD-DOT device (LUMO, Gowerlabs [37]) on the motor cortex. The subject performed four common movement types: nodding the head, shaking the head, raising the eyebrows, and moving the jaw. The experiment started with a 30-second motion-free baseline period, followed by a single movement within 2 seconds per trial, and an 8-second rest to isolate the MAs of each motion. Each type of movement was repeated 25 times.

The raw DOT data were converted to OD using the 30-second baseline. The OD of each channel spanning 4 seconds before and after each motion was extracted for analysis. The extracted MAs caused by the four different motions exhibited no distinctive differences in shape and were consistently represented as linear combinations of a spike-shaped component followed by a step-shaped component, with both components having either positive or negative amplitudes.

**Fig. 3.**
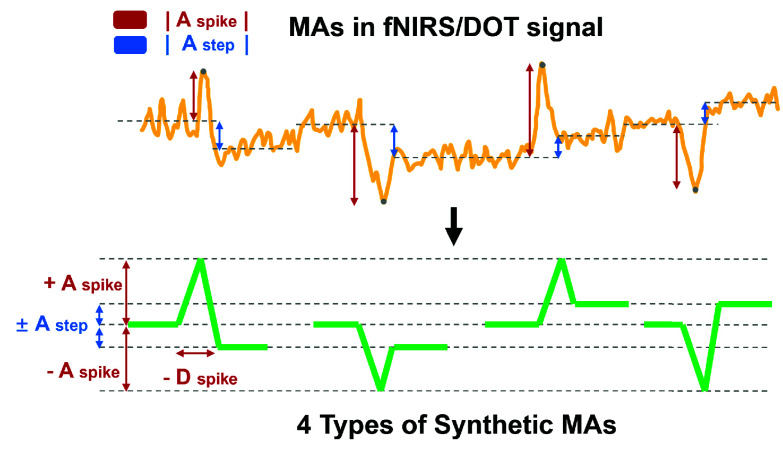
Schematic of the MA modeling in fNIRS/DOT signals, illustrating four types of synthetic motion artifacts (MAs) caused by active movement.

The synthesized MAs were modeled accordingly. The amplitude of the step-shaped MA was defined as the absolute difference between the average OD before and after the motion, ranging from 2.38 to 8.25 times the standard deviation of the resting-state data. The peak amplitude of the spike-shaped MA was defined as the difference between the average OD before the motion and the peak (or trough) during the motion, with values ranging from 25.53 to 46.93 times the standard deviation of the resting-state data. The durations of the spike-shaped ranged from 0.40 to 1.05 seconds, with the peak consistently occurring at the midpoint of the duration. The polarity (positive or negative) of both components was randomly assigned and combined. The feature extraction and modeling of the MAs from the experiment ensure that the simulated MAs accurately replicate the real-world MA patterns. The overall process of MA modeling is illustrated in Fig. 3.

### C. Deep Learning Based MA Correction Model

#### 1) MA-Contaminated Dataset Generation:

In this study, MA-contaminated data were generated by introducing synthetic MA into clean OD signals. Channel pruning was first performed based on predefined criteria, including power range and SNR, drawn from the established fNIRS processing toolbox, HomER2 [38]. Active channels were identified if their light intensity in dB fell within the power range of 85-125 dB, and if their SNR was more than 17.8 dB. Then the light intensity of all the active channels was converted to OD using (3). The OD values for active channels were then rescaled using (6), where the mean value and standard deviation of each channel were calculated for standardization.

\begin{equation*} OD_{standardized}\mathrm {=}(OD-mean(OD\mathrm {))/}std\left ({{ OD }}\right ) \tag {6}\end{equation*}

The standardized OD data across all channels were segmented into windows of 100 time points, corresponding to approximately 15 seconds at a 6.67 Hz sampling rate. For each window, one synthetic MA described in Section III.B was generated and added to the original OD data. The amplitude and duration of the synthetic MAs were randomly selected within the specified ranges outlined in Section III.B. After introducing the synthetic MAs, the contaminated OD signals were rescaled to their original scale by reversing the standardization process. The windows with added MA noise, along with the corresponding clean data, were stored as training samples for the deep learning model.

#### 2) DAE Modelling:

**Fig. 4.**
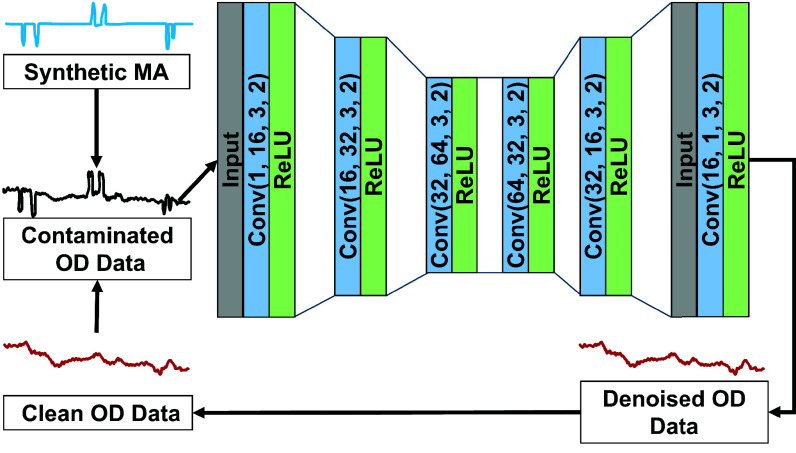
Architecture of the proposed DAE model for MA correction. The training dataset was synthesized by adding synthetic MAs to clean OD data.

**Fig. 5.**
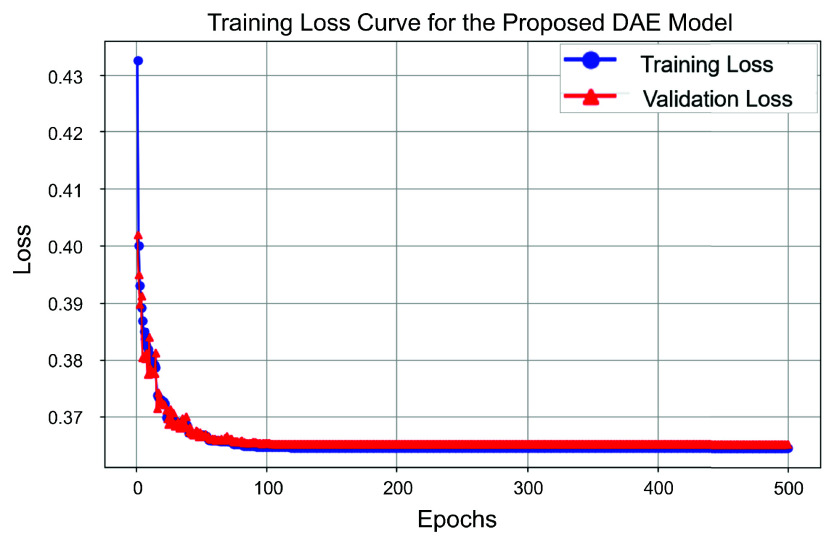
Training and validation loss curves for the proposed DAE model over 500 epochs.

A DAE model was designed and implemented using PyTorch [39] in Python 3.12 for MA correction (Fig. 4). The encoder is composed of three consecutive 1D convolutional layers with kernel sizes of 3, strides of 2, and ReLU activations, progressively reducing the input dimensions. The number of channels increases from 1 to 16, 32, and finally 64. The decoder mirrors the encoder with three transposed convolutional layers, using similar kernel sizes and strides. The current architecture was optimized considering the duration (~15 seconds) of each fNIRS data window in this study and the trade-off between processing time and performance in real-time applications. Spike-shaped motion artifacts in this study last from 0.4 to 1.05 seconds, corresponding to approximately 3 to 7 timepoints at a 6.67 Hz sampling rate. Adjustments to kernel size, stride, and activation functions were made to achieve optimal performance. Additionally, fewer layers were tested but significantly degraded the model’s effectiveness. The loss function used was the mean squared error (MSE) between the non-contaminated data and the model output. The model was trained for 500 epochs with a learning rate of 0.0005, using the Adam optimizer. The training loss curve for the DAE model is shown in Fig. 5.

#### 3) Real-Time Application of DAE Model:

As the DAE model for MA correction operates within a fixed time window, modifications are necessary to enable its use in real-time settings. To achieve real-time processing, a sliding window scheme was implemented in our design. A 15-second time window is applied to the data across all channels, with the window sliding forward by 3 seconds at each step. This allows every 3-second segment of data to be processed by the DAE model with five repetitions and varying length of adjacent data. The final output for each segment is calculated as the average for these five repetitions. Newly streamed data are initially visualized as they arrive, and as the window continues to slide, the results for that segment are updated based on the averaged model outputs.

### D. Real-Time Preprocessing System

#### 1) Calibration/Initialization:

In developing a real-time preprocessing system for fNIRS, a key challenge is obtaining the requisite information for conventional preprocessing, which includes optode positions, source-detector separations and channel baseline. To address this, we employed a calibration phase before the implementation of the real-time system. During this stage, subjects are asked to remain still for approximately 30 seconds to record the raw fNIRS/DOT data. For the registration, the positions of optode and cranial landmark can also be acquired either directly on the subject during the calibration phase or alternatively, on a phantom model equipped with the device before the calibration, with the data then stored for future use.

Once the calibration process is complete, a MATLAB script computes the mean value and standard deviation for the raw light intensity of each channel, which is then used for OD conversion using (3). The means and standard deviations of the OD from all channels are also calculated for the MA correction step in the real-time preprocessing. Subsequently, channel pruning is performed based on the predefined criteria described in Section III-A. A list of active channels is acquired based on the pruning criteria. Furthermore, the system calculates and stores the source-detector separations of each channel based on optode positioning, categorizing them into long and short channels. This categorization can potentially be utilized for short-channel regression [40]. Additionally, bandpass filtering is employed to eliminate physiological noise. A third-order Butterworth low-pass filter (0.5 Hz) and a fifth-order Butterworth high-pass filter (0.05 Hz) are used, with filter parameters calculated via the MakeFilter function in MATLAB [41]. The mean value and standard deviation of light intensity and OD, source-detector separations, the active channel list, and filter parameters are all saved and can be reloaded into the real-time preprocessing system.

#### 2) Real-Time Preprocessing System Pipeline:

**Fig. 6.**
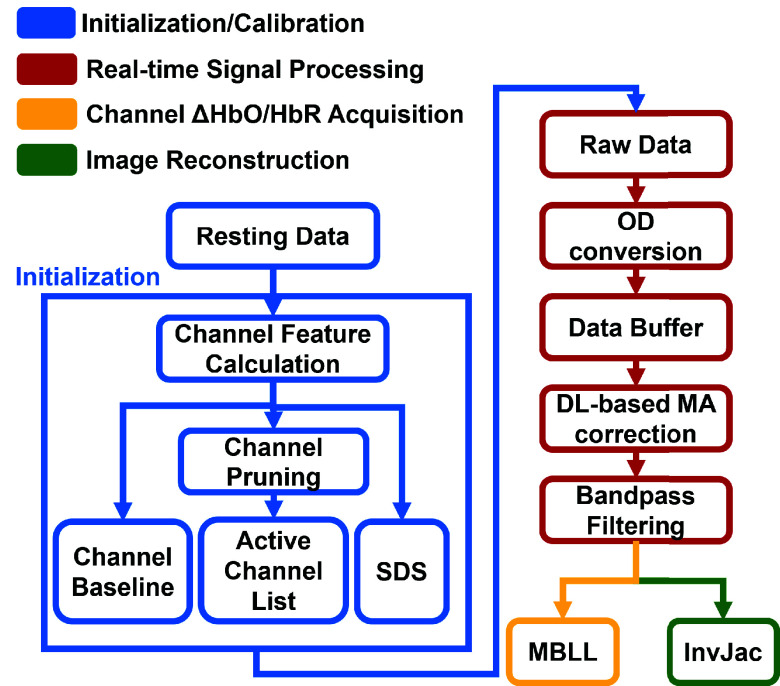
Schematic of the proposed real-time processing system. Following a resting-state initialization, streaming fNIRS/DOT data is processed in real-time. The system enables channel-wise hemodynamic information acquisition or 3D image reconstruction.

The real-time preprocessing system is developed using MATLAB on an open-source platform, Lab Streaming Layer (LSL) [42], for streaming data from fNIRS devices (Fig. 6). The raw, multi-channel fNIRS data from the wearable device are streamed to LSL sample by sample using the lsl_inlet() function [42]. Channel information calculated during the calibration process is loaded as parameters. For each sample, the multi-channel light intensity data are first converted to OD using (3), with the average intensities $\bar {I}$ across all channels obtained from the calibration parameters. The OD values for all channels are then globally rescaled before real-time MA correction using (6), where the global mean value and standard deviation of OD are also derived from the loaded calibration parameters. After this, a local standardization is performed by rescaling the current window based on the mean value and standard deviation of the previous MA corrected window. This step ensures the input of DAE MA correction model remains close to a normalized distribution, as the model produces more stable outputs when processing standardized data.

\begin{equation*} OD_{normalized}=\frac {OD-mean\left ({{OD_{calibration} }}\right )}{OD_{calibration}} \tag {7}\end{equation*}

After the MA correction described in Section III.B, the denoised data is rescaled and recovered using the previously determined global and local standardization parameters. It is then filtered using MATLAB filter function with the parameters loaded from calibration. The filtered channel ODs can then be utilized for both channel-level hemodynamics calculation and 3D image reconstruction, which integrates information from multiple channels to provide a comprehensive representation of 3D brain hemodynamics. For the channel-level hemodynamics calculation, the Modified Beer-Lambert Law shown in (6) is applied.

As $\mathrm {\Delta }OD$ is compared with the baseline OD, the output filtered OD of this system can be directly applied to calculate the relative changes of the HbO and HbR in each channel. These channel-level hemodynamic data can be streamed in real-time to enable modulation of brain activity in NFB applications or combined with feature extraction techniques for signal classification in BCI.

For image reconstruction of the 3D brain hemodynamics, the Jacobian matrix is computed using Toast++ [43], based on the registration information from an adult head phantom. The inverse problem is pre-solved using zeroth-order Tikhonov regularization, with the hyper parameter $\lambda $ set to 0.01. In the real-time system, the inverse Jacobian matrix is truncated to include only the active channels and directly multiplied with the filtered OD, as shown in III). The result $\mathrm {\Delta }x$ (changes in absorption coefficient within the brain tissue) can be used to generate volume-wise plots for 3D brain hemodynamics, providing direct feedback to the user in NFB applications. Alternatively, data from specific brain regions can be streamed in real-time for signal classification in BCI systems.

### E. Validation Experiment

To validate the performance of the DAE model for MA correction, we tested it on the test set from the 36-module, whole-head HD-DOT data. To assess the real-time capability of the processing system, we tested it on the 12-module HD-DOT data collected from the motor cortex during the motor imagery experiment. Both datasets are described in Section III-A. We manually contaminated the dataset as described in Section III-D and processed it through the proposed real-time system, comparing the results with those from an offline processing system that employed various MA correction methods.

The offline processing pipeline included channel pruning, $O D$ conversion, MA correction, band-pass filtering, and image reconstruction based on the default source-detector layout, including Jacobian and inverse Jacobian calculations. For the channel baseline during OD conversion, we used the average light intensity across the entire experiment of each channel for both real-time and offline processing to allow for a better comparison of MA correction methods. We compared the MSE and correlation coefficient (CC) between the corrected OD signals and the original clean OD signal across different MA correction methods, including MARA, tPCA, k-wavelet, splineSG, TDDR and the proposed real-time DAE model [2025]. The offline MA correction methods were implemented using the homER2 toolbox [38] and the TDDR function [24], utilizing the functions hmrMotionArtifactByChannel with hmrMotionCorrectSpline (spline interpolation, $\mathrm {tMotion}=0.5$, tMask=2 STDEVthresh=20, AMPthresh=0.5, pSpline=0.99), hmrMotionCorrectPCArecurse (tPCA, nSV=0.97, maxIter=3), hmrMotionCorrectKurtosisWavelet (k-wavelet, threshold =3.3), hmrMotion CorrectSplineSG (SplineSG, p=0.99, FrameSize_sec=10) and TDDR function. The evaluation of system performance in image reconstruction was conducted by comparing block-averaged 3D images that indicate hemoglobin concentration changes in gray matter. These reconstructions utilized MA-corrected data from various correction methods to assess the effectiveness of the image reconstruction process.

## IV. Results

### A. Evaluation of Processing Accuracy

**Fig. 7.**
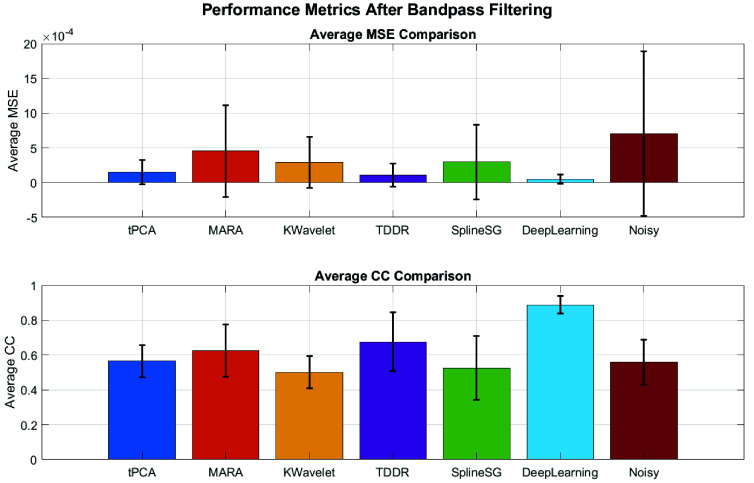
Average Mean Square Error (MSE) and Correlation Coefficient (CC) with the original clean signal for different MA correction techniques after bandpass filtering. The proposed deep-learning-based, DAE model for real-time MA correction achieved the lowest MSE and highest CC.

The whole-head, multi-task HD-DOT data, which was manually contaminated with MAs, was used to assess the performance of the proposed MA correction method. The performance results of the different MA removal methods are shown in Fig. 7. The evaluation compared various MA correction techniques, and the proposed deep-learning-based, DAE model for real-time MA correction achieved the lowest OD MSE of $0.0001\pm 0.0001$ and the highest CC of $0.8881\pm 0.0493$ across all methods after the bandpass filtering.

**Fig. 8.**
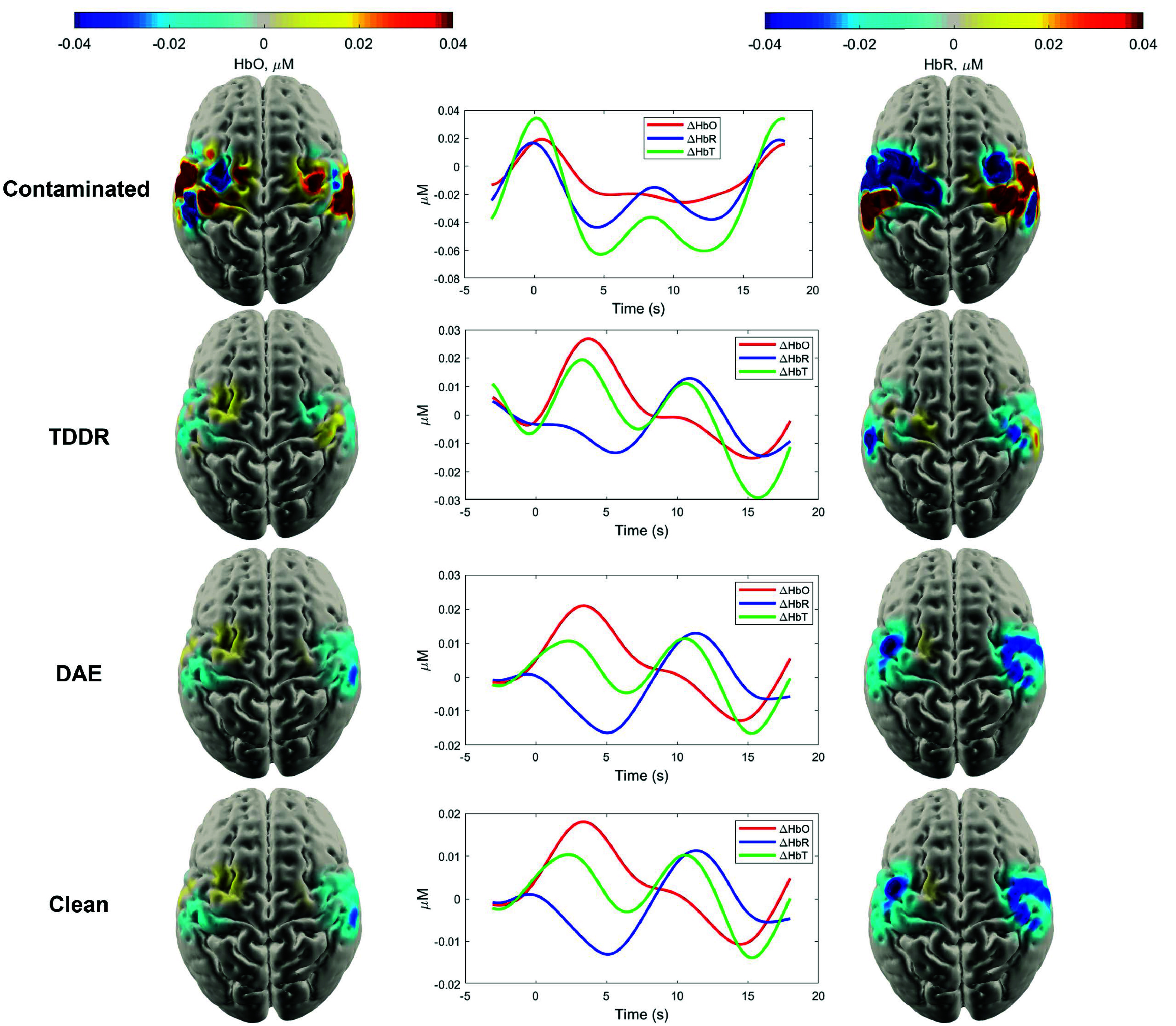
Block-averaged 3D images of $\triangle $HbO and $\triangle $HbR on the gray matter from contaminated, TDDR-corrected, DAE-corrected, and clean data for right-hand MI, including the block-averaged hemodynamic response function (HRF) at the node with the highest response in the clean image.

**Fig. 9.**
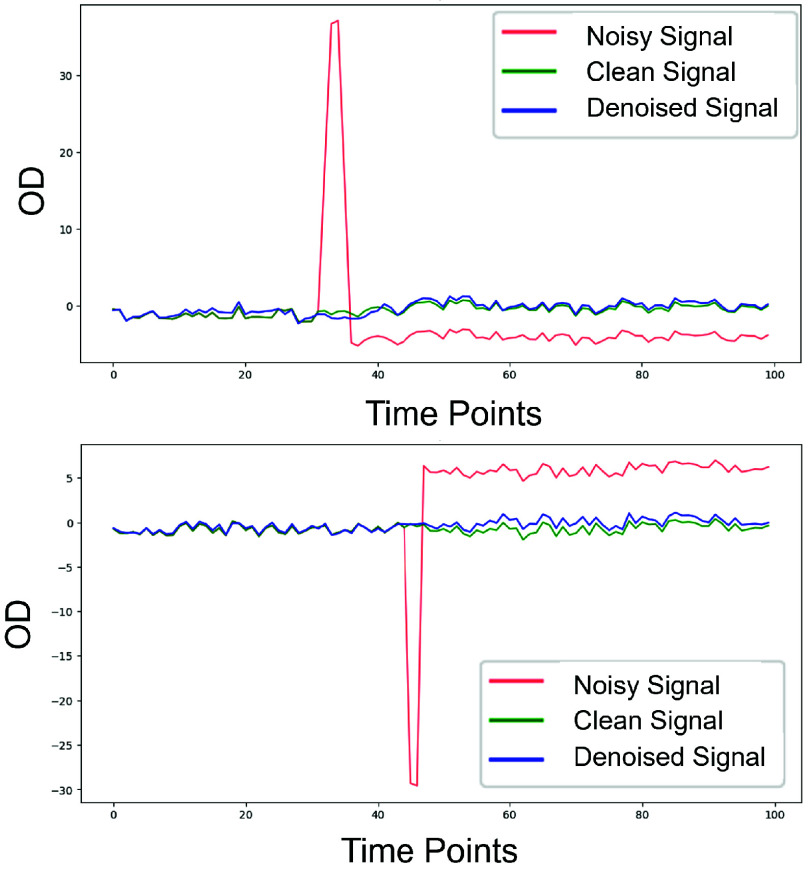
Illustration of the DAE-based MA correction method applied to a window of data from a specific channel. The figure compares noisy signals, clean signals, and denoised signals before and after the correction process.

Fig. 8 and Fig. 9 demonstrates the effectiveness of the DAE model in correcting MAs. Fig. 9 shows the comparison between the noisy signal, clean signal, and denoised signal. This visualization highlights the DAE model’s effectiveness in reducing MAs while preserving the integrity of original signal.

The block-averaged image reconstruction results obtained from MA corrected data using the TDDR and DAE methods, as well as the original clean data and contaminated data, are presented in. The average hemodynamic response function of the four reconstruction results at the node with the highest response in clean image is also displayed in Fig. 8. The reconstructed image from the DAE output closely aligns with the functional mapping of the original clean data. In contrast, the contaminated data is primarily affected by MAs, and the TDDR result also exhibits partial contamination by MAs.

### B. Evaluation of Processing Speed

**Fig. 10.**
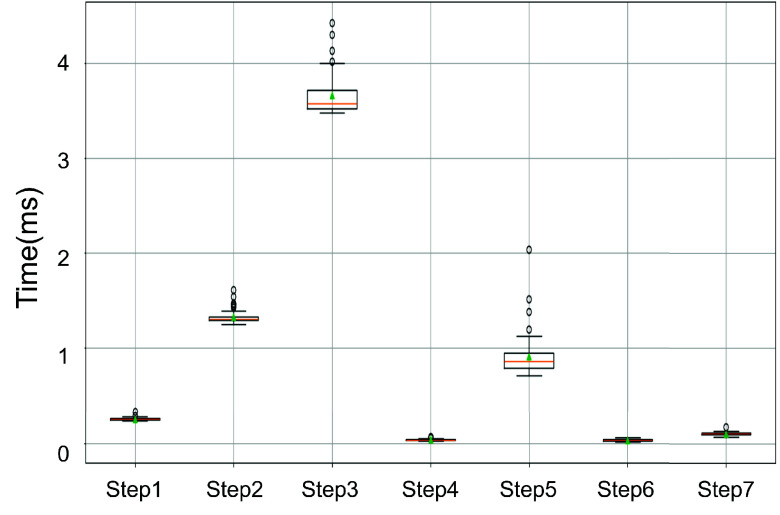
Box plot showing the distribution of processing times for each preprocessing step applied to 756 active channels of HD-DOT data.

The processing speed of the system was evaluated on the HD-DOT data over the motor cortex from the motor imagery experiment. The average processing time for each step outlined in Section III-D, when applied to 756 active channels, is presented in Table I. Additionally, a box plot illustrating the distribution of the processing times for each step is provided in Fig. 10. The total average processing time per sample is 6.33 ms. Among the steps, OD normalization was the most time-consuming step (3.66 ms). Despite this, the system is capable of completing the preprocessing within the time interval between two consecutive samples (given the current sampling rate of 12.5 Hz), demonstrating its ability to process multi-channel DOT data in real-time.

**TABLE I:** Averaged Processing Time of Each Step in the Proposed Real-Time Processing System

Processing Step	Averaged Processing Time (ms)
1. Active Channel Extraction	0.26
2. OD Conversion	1.33
3. OD Normalization	3.66
4. Denoising with DAE Model	0.04
5. Reversing Normalzation	0.91
6. Bandpass Filtering	0.03
$7.~\Delta $HbO& $\Delta $HbR Concentration Conversion	0.10
Total Processing	6.33

## V. Discussion and Future Work

The DAE-based MA correction method, integrated into the proposed real-time processing and imaging system, outperforms traditional methods. The proposed system enables low-latency preprocessing of fNIRS/DOT data, delivering real-time channel-wise oxygenation information and 3D functional imaging. Its capability to handle high-channel data (over 756 channels) in real time provides a promising solution for systems like DOT and HD-DOT to efficiently acquire 3D high-resolution functional images. These advantages could support fNIRS/DOT to accurately monitor the neural activity of targeted brain regions which are valuable for both BCI and NFB applications.

For BCI applications, the spatial localization capabilities of fNIRS/DOT facilitate the classification of mental activities associated with different brain regions, enhancing the ability to distinguish subtle activation patterns. This enables precise command differentiation, such as distinguishing between motor imagery of the foot and the hand [44], [45]. Additionally, it can monitor mental workload to better evaluate the cognitive performance of users when using the BCI [46]. It can also be used to assess rehabilitation progress by observing neural activity throughout the entire rehabilitation training process [47]. For NFB applications, feedback on brain activation specific to disorders and dysfunctions can be given to the user. This allows them to self-regulate abnormal brain processes towards a desired state, helping to reduce neurological and psychiatric symptoms [5].

The real-time processing capability of the proposed system could also enhance the integration of DOT and HD-DOT with EEG-based systems in BCI and NFB, compensating in part for the spatial resolution limitations of EEG. This integration could not only improve the classification accuracy of neural patterns in BCI but also provides more direct 3D mapping of brain activation to feedback to users in NFB [2], [48].

Moreover, the DAE-based MA correction demonstrates superior performance in eliminating MAs while preserving the integrity of the original data. The sliding window strategy enables the DAE-based MA correction to operate in a real-time processing format, maintaining high performance with low latency. These advantages make the DAE-based real-time MA correction method promising for DOT and HD-DOT system.

Despite the low delay of the system in processing data samples, intrinsic delays remain due to the window processing logic required for the real-time application of the DAE model. To address this, implementing an efficient MA detection mechanism to activate the DAE model only when artifacts are detected could optimize system speed and enhance processing accuracy [49]. This selective processing approach would reduce computational load and further minimize latency.

This study serves as an initial exploration into deep-learning-based, real-time signal processing for DOT and HD-DOT systems. Further validation through experimental trials on real-world, motion-contaminated data is necessary to support and refine the current design. Future work will focus on improving the performance of the DAE model for MA correction. This will involve expanding the training dataset by including a more diverse group of subjects performing active MA experiments and refining the extraction of MA features across this broader cohort. These enhancements aim to enable the model to address a wider variety of MAs.

Incorporating spatial information, such as the source and detector positions within DOT and HD-DOT data, into the deep learning model could potentially enhance accuracy and reduce latency in multi-channel MA correction for these systems. Additionally, real-time implementation of short-channel regression techniques [40], utilizing methods like adaptive filtering [50], could further improve functional mapping capabilities of DOT and HD-DOT systems.

Finally, integrating an efficient real-time MA detection technique before correction could significantly reduce computational demands by avoiding unnecessary processing of non-contaminated data.

## VI. Conclusion

This study presents a real-time, multi-channel processing system for fNIRS/DOT, integrating a DAE-based deep learning model for MA correction and a pre-calculated inverse Jacobian matrix for rapid 3D functional imaging. When processing high-channel HD-DOT data, the system demonstrates excellent performance, effectively correcting MAs, preserving data integrity, and maintaining low latency. This work marks an encouraging first step toward fully unblocking the potential of DOT and HD-DOT technologies to expand their applications in BCI/NFB for rehabilitation, neurological diseases, and behavior disorders.
